# Medical experience of family members of children in pediatric infusion room: a cross-sectional survey

**DOI:** 10.3389/fped.2025.1674036

**Published:** 2025-10-14

**Authors:** Dan Zhao, Yong Liu, Jing Zhang, Yan Wang, Dan Chen

**Affiliations:** Department of Nursing, Children’s Hospital of Nanjing Medical University, Nanjing, Jiangsu, China

**Keywords:** medical experience, family, children, pediatric, care, nursing, clinical

## Abstract

**Background:**

Enhancing the medical experience of children and their family members constitutes a crucial component of clinical management. This study aims to analyze the current status of the medical experience among family members of children in pediatric infusion rooms, thereby providing evidentiary support for the management of clinical health services.

**Methods:**

We included children who received treatment in the pediatric infusion room of our hospital and their family members from February 2025 to May 2025. The questionnaire for family members’ medical experience was used for survey.

**Results:**

A total of 546 family members were included. The mean score for the medical experience of these family members was 3.95 ± 1.07. Correlation analysis demonstrated that several variables were significantly associated with the medical experience scores: age of the family members (*r* = −0.602), their relationship with the child (*r* = 0.557), educational level (*r* = −0.577), the child's age (*r* = 0.622), the child's birth order (*r* = 0.584), and the child's type of medical insurance (*r* = 0.620). Further multivariate linear regression analysis confirmed that these six variables—age of the family member, relationship with the child, educational level, child's age, child's birth order, and child's medical insurance type—were independent influencing factors of the medical experience. The regression model accounted for 56.6% of the variance in the medical experience scores.

**Conclusion:**

The medical experience of family members of children in the observation and infusion department of children's hospitals still needs to be further improved. Medical workers should take appropriate measures against the relevant influencing factors to enhance their medical experience.

## Introduction

Child health, as the cornerstone of national health, its security level is directly related to the improvement of population quality and the sustainable development of society (1). Within the pediatric medical service system, intravenous therapy has become an important means for the treatment of common childhood diseases due to its characteristics of rapid effect and wide application range. The pediatric infusion room, as the core place for implementing this treatment, its service quality is closely related to the medical experience of children's family members (2). As the main companions and decision-makers in the process of children's medical treatment, the subjective feelings of family members during a series of processes in the infusion room, such as registration, waiting for treatment, treatment, and environmental adaptation, not only reflect the degree of humanistic care in medical services, but also affect the family members’ cooperation with medical behaviors and the harmony of doctor-patient relationship (3, 4).

With the continuous deepening of the medical system reform, patient experience has become one of the core indicators to measure the quality of medical services (5). At present, the research on adult medical experience has formed a certain system, but there are still deficiencies in the research on family members’ experience in the special scenario of pediatric infusion rooms (6, 7). On the one hand, the pediatric infusion room has the characteristics of a large number of people, frequent crying of children, and concentrated anxiety of family members, so the medical experience of family members presents more complex characteristics (8). On the other hand, existing studies mostly focus on the description of problems in a single link, lacking a systematic evaluation of the current situation of experience and in-depth analysis of influencing factors, which leads to the lack of scientific basis for the formulation of targeted improvement measures (9). In this context, it has become an important issue to improve the quality of pediatric medical services and build a harmonious doctor-patient relationship to deeply explore the current situation of medical experience of family members in pediatric infusion rooms, identify key influencing factors and put forward optimization countermeasures.

Therefore, this study aims to analyze the current situation and influencing factors of medical experience of family members of children in pediatric infusion rooms, so as to provide reliable evidence support for clinical nursing and management.

## Methods

This study adopts a cross-sectional survey design, and the research protocol has been reviewed and approved by the Children's Hospital of Nanjing Medical University's Ethics Committee (Approval No.: 202509003-1). All participants signed written informed consent forms, and all collected information will be used exclusively for this survey.

The study was conducted in the infusion room of a tertiary children's hospital in Jiangsu province, China. This hospital is one of the leading pediatric medical institutions in the region. The infusion room has a total of 295 observation beds (chairs) for children receiving intravenous infusions. On average, it receives 1,500–1,900 children for intravenous infusions daily, with the number sometimes reaching a peak during flu seasons or disease outbreaks. The medical staff—to—patient ratio in the infusion room is approximately 3:100. However, during peak patient capacity, this ratio becomes strained, as the number of patients far exceeds the normal load. Despite the challenges, the nursing team in the infusion room is well—trained, with a one—time intravenous puncture success rate of over 95%. Nevertheless, factors such as the high patient—to—staff ratio during peak times may still impact the overall medical experience of family members. Understanding these characteristics of the infusion room environment is crucial for assessing the feasibility of the proposed solutions and conclusions in the study.

This study included children who received treatment in the pediatric infusion room of our hospital and their family members as research subjects, with the time range from February 2025 to May 2025. The inclusion criteria were set as follows: family members who had a blood relationship with the child and were aged 18 years or older; the family members had accompanied the child to complete treatment in the pediatric infusion room of our hospital; and the family members had fully understood the content of this study and voluntarily agreed to participate. The exclusion criteria included: family members of children in the special needs clinic; family members who had clearly expressed their unwillingness to participate in this survey.

Relevant information of the children and their family members was collected through the medical record system and communication, specifically including the following items: age, gender, relationship with the child, whether it was the first visit to this hospital, place of residence, educational level, employment status, monthly per capita household income (in Yuan), the child's age (we chose 10 years as the cutoff based on developmental psychology and clinical practice, which mark the transition from childhood to early adolescence. This transition is typically associated with increased communication abilities, greater autonomy, evolving coping mechanisms, and changes in parental involvement and anxiety levels.), the child's gender, the child's birth order, and the child's type of medical insurance.

In this study, the medical experience of family members of children was evaluated using a questionnaire for family members’ medical experience that had been developed and validated in previous study (10). The questionnaire consists of 26 items across three dimensions: Medical Process, Medical Behavior, and Medical Environment, and adopts a Likert 5-point scoring method (1–5 points). A higher score indicates a better medical experience of the respondents. Specifically, a mean score of <3 points indicates a poor medical experience, 3–4 points indicates a moderate medical experience, and >4 points indicate a good medical experience. Additionally, the Cronbach's α coefficient of this scale is 0.821, demonstrating good reliability and validity (11).

In the data collection phase of this study, a strictly standardized procedure was followed. All research team members received systematic and specialized training, which covered the interpretation of core questionnaire content, survey ethical norms, communication skills, and handling of unexpected situations, ensuring the consistency and professionalism of the survey implementation. This survey adopted paper questionnaires, which were distributed by specially assigned personnel who had obtained unified qualification certification. During the survey implementation, at an appropriate time when family members accompanied the children to receive infusion treatment, investigators explained the research purpose, questionnaire filling requirements, and confidentiality principles to eligible family members. Questionnaires were distributed on-site after obtaining their consent. The questionnaires were filled out and collected on-site to minimize information omission and deviation to the greatest extent possible. To ensure the objectivity and authenticity of the data, all investigators passed unified training and assessment. It was clearly stipulated that during the answering process, they should only provide standardized explanations for questionnaire items that family members had questions about, without exerting any guidance or interference on the family members’ subjective judgments. This operational model not only ensured that family members could accurately understand the connotation of evaluation indicators but also fully respected their independent judgments based on their own medical experience.

In this study, SPSS 25.0 statistical software was used for data collation and analysis to ensure the standardization of the statistical process and the reliability of the results. For the count data involved in the study, rates (%) were used for description to reflect the distribution of different categorical indicators; for the measurement data, the mean ± standard deviation was used to present the central tendency and dispersion of the data. At the univariate analysis level, appropriate statistical methods were selected according to the data type and research purpose: the *t*-test was used for the comparison of measurement data between two groups; ANOVA one-way analysis of variance was used for the comparison of measurement data among multiple groups to explore the differences in medical experience scores among different groups. To clarify the degree of correlation between medical experience scores and the personal characteristics of the research subjects, Pearson correlation analysis (applicable to data conforming to a normal distribution) or Spearman correlation analysis (applicable to data not conforming to a normal distribution) was adopted. In the multivariate analysis, the multiple linear regression analysis method was used, and multiple factors that might affect the medical experience of the family members of the children were included in the model to control the interference of confounding factors, thereby more accurately identifying the key variables that have a significant impact on the medical experience. The significance level of this study was set at *α* = 0.05.

## Results

A total of 546 family members of children receiving treatment in the pediatric infusion room were enrolled in this study. The mean age of the family members was 38.85 ± 9.40 years. Among them, 55.86% were female, with the majority being the children's mothers. In terms of residential distribution, 69.78% were urban residents, and 73.63% were currently employed. The mean age of the children was 9.66 ± 3.01 years. The details of characteristics of participants are presented in Table 1.

**Table 1:** Characteristics and univariate analysis of medical experience of family members of children in pediatric infusion room (*n* = 546).

Characteristic	Cases (%)	Mean medical experience score	t/F	*p*
Age			2.445	0.018
<30	116 (21.25%)	4.14 ± 1.05		
30–60	306 (56.04%)	3.96 ± 1.07		
>60	124 (22.71%)	3.10 ± 1.19		
Gender			1.280	0.109
Male	241 (44.14%)	4.03 ± 1.01		
Female	305 (55.86%)	3.90 ± 1.12		
Relationship with the child			1.847	0.006
Father	157 (28.75%)	4.07 ± 1.15		
Mother	254 (46.52%)	3.93 ± 1.03		
Grandparents	106 (19.41%)	3.77 ± 1.16		
Others	29 (5.31%)	3.96 ± 1.04		
First visit to this hospital			1.534	0.074
Yes	140 (25.64%)	3.88 ± 1.15		
No	406 (74.36%)	3.98 ± 1.04		
Place of residence			1.260	0.117
Urban area	381 (69.78%)	3.99 ± 1.20		
Rural area	165 (30.22%)	3.89 ± 1.13		
Educational level			1.775	0.040
Postgraduate and above	24 (4.40%)	4.10 ± 1.05		
Undergraduate/College	188 (34.43%)	4.02 ± 1.17		
Senior high school	243 (44.51%)	3.90 ± 1.24		
Junior high school	54 (9.89%)	3.85 ± 1.04		
Primary school and below	37 (6.77%)	3.66 ± 1.19		
Employment status			1.268	0.143
Employed	402 (73.63%)	3.99 ± 1.17		
Unemployed	56 (10.25%)	3.90 ± 1.29		
Retired	88 (16.12%)	3.91 ± 1.14		
Monthly per capita household income (Yuan)			1.330	0.126
<5,000	354 (64.84%)	3.92 ± 1.14		
≥5,000	192 (35.16%)	3.99 ± 1.05		
Child's age			1.288	0.019
<10	376 (68.86%)	3.90 ± 1.17		
≥10	170 (31.14%)	4.06 ± 1.09		
Child's gender			1.005	0.180
Male	289 (52.93%)	3.92 ± 1.20		
Female	257 (47.07%)	3.98 ± 1.15		
Child's birth order			1.294	0.007
First child	204 (37.36%)	3.60 ± 1.01		
Second child and above	342 (62.64%)	4.08 ± 1.14		
Child's medical insurance type			1.133	0.034
Resident medical insurance	483 (88.46%)	3.97 ± 1.15		
Commercial insurance	24 (4.40%)	4.09 ± 1.03		
Self-payment	30 (5.49%)	3.43 ± 1.26		
Others	9(1.65%)	3.85 ± 1.07		

The mean score of medical experience among family members of children in the pediatric infusion room was 3.95 ± 1.07, indicating that their overall medical experience was moderate, with room for improvement. Among the dimensions, the score for “Medical Process” was the lowest. The details of medical experience score of family members of children in pediatric infusion room are presented in Table 2.

**Table 2:** The medical experience score of family members of children in pediatric infusion room.

Dimension	Number of items	Mean score
Medical process	12	3.89 ± 1.15
Medical behavior	10	3.99 ± 1.09
Medical environment	4	3.97 ± 1.21
Total	26	3.95 ± 1.07

There were statistically significant differences in medical experience scores among family members with different ages, relationships with the child, educational levels, as well as among those with children of different ages, birth orders, and medical insurance types (all *p* < 0.05).

Correlation analysis indicated that age (*r* = −0.602), relationship with the child(*r* = 0.557), educational level(*r* = −0.577), child's age (*r* = 0.622), child's birth order(*r* = 0.584), child's medical insurance type(*r* = 0.620) were correlated with medical experience score of family members of children in pediatric infusion room. The results of correlation analysis on the characteristics and medical experience score of family members of children in pediatric infusion room are presented in Table 3.

**Table 3:** Correlation analysis on the characteristics and medical experience score of family members of children in pediatric infusion room.

Variables	r	*p*
Age	−0.602	0.014
Gender	0.165	0.092
Relationship with the child	0.557	0.043
First visit to this hospital	0.112	0.219
Place of residence	0.106	0.140
Educational level	−0.577	0.034
Employment status	0.125	0.109
Monthly per capita household income (Yuan)	0.118	0.067
Child's age	0.622	0.024
Child's gender	0.107	0.098
Child's birth order	0.584	0.041
Child's medical insurance type	0.620	0.025

Multiple linear regression analysis indicated that age, relationship with the child, educational level, child's age, child's birth order, child's medical insurance type were the influencing factors of medical experience of family members of children in pediatric infusion room (all *p* < 0.05). The regression model showed good fitting performance, with *R*^2^ = 0.592 and adjusted *R*^2^ = 0.566, indicating that the model could explain 56.6% of the variation in the medical experience scores of the family members. The F-statistic of the model was 48.264 (*p* < 0.001), suggesting that the overall regression equation was statistically significant. The results of the multiple linear regression analysis identifying factors influencing the medical experiences of family members of children in the pediatric infusion room are presented in Table 4.

**Table 4:** Multiple linear regression analysis on the influencing factors of medical experience of family members of children in pediatric infusion room.

Variable	Unstandardized β	SE	Standardized β	t	*p*
Constant	−28.492	3.103	–	−9.126	0.012
Age	−0.811	0.180	−0.303	−3.850	0.023
Relationship with the child	0.784	0.209	0.250	2.108	0.016
Educational level	−0.812	0.174	−0.324	−3.856	0.003
Child's age	0.895	0.112	0.370	2.995	0.034
Child's birth order	0.712	0.120	0.229	3.009	0.021
Child's medical insurance type	0.862	0.167	0.320	2.704	0.008

*R*^2^ = 0.592, adjusted *R*^2^ = 0.566, F = 48.264, *p* < 0.001.

## Discussion

Our findings indicate that the overall medical experience of family members in the pediatric infusion room is moderate, suggesting current services meet basic needs but leave substantial room for improvement, consistent with prior studies noting pediatric care experiences are “above average but lack refinement in details” (12). Critically, the “medical process” dimension received the lowest scores, identifying it as the primary driver of dissatisfaction and the most impactful leverage point for improvement. As the “first point of contact,” this process encompasses registration, waiting, consultation, examination, and payment, whose efficiency directly influences both time costs and psychological well-being. Viewed through the lens of service continuity, pediatric infusion forms a sequential chain (“registration—waiting—consultation—examination—infusion—medication pickup”) where congestion at any node can trigger a negative cascade; inferred issues such as mismatches between appointment and actual consultation times or prolonged waits for results likely compound dissatisfaction (13). Moreover, family members’ “dual-role pressure” (as caregivers and service recipients) amplifies frustration when delays occur while comforting distressed children, consistent with the “time perception bias” theory, where anxiety stretches subjective wait times (14, 15). Consequently, interventions should prioritize process optimization that simultaneously enhances objective efficiency (e.g., streamlining workflows and improving coordination) and manages subjective perceptions (e.g., providing timely information and emotional support), thereby directly addressing our key finding to most effectively elevate the overall experience.

Multiple linear regression analysis shows that the older the family members and the lower their educational level, the worse their medical experience, which is consistent with research conclusions on the adaptability of demographic characteristics to medical services. Elderly family members may be unfamiliar with hospital information systems (such as self-service registration and electronic report inquiry), increasing operational difficulties and time costs; family members with lower educational levels may have obstacles in understanding medical advice and filling out forms, leading to a decline in their sense of control over services and thus lowering their experience scores (16). This result suggests that medical services need to pay attention to “differentiated needs.” Family members of different ages and educational backgrounds have significant differences in their needs for service forms: elderly family members rely more on manual guidance, while those with lower educational levels need simpler information transmission methods (17). Ignoring such differences may lead to a mismatch between service supply and demand, exacerbating the gap in experience.

The study found that the younger the children, the more they are first-born, and the worse the medical experience of their family members, which is closely related to the “care burden theory” in pediatric nursing. Younger children (such as infants and toddlers) have limited expressive abilities and often show discomfort through crying, increasing the pressure on family members to comfort them (18); at the same time, younger children have faster changes in their conditions, and their family members have stronger concerns about the safety of medical operations, which can easily amplify deficiencies in services due to tension (19).

Family members of first-born children, due to their lack of experience in child-rearing and medical treatment, pay more attention to the details of medical services and have more idealized expectations for “ideal services.” When the actual services do not meet expectations (such as insufficient explanation of nurses’ operations), their dissatisfaction is more likely to be aroused. This is consistent with relevant research findings — anxiety scores of first-time parents in pediatric medical scenarios are significantly higher than those of non-first-time parents, and anxiety levels are negatively correlated with medical experience (20).

Family members with self-funded medical care have a worse medical experience, a result that highlights the potential impact of economic factors on the evaluation of medical services. Self-funded patients need to bear all medical expenses directly and are more sensitive to “cost-value matching": when waiting time is too long or service details are insufficient, they are prone to the perception of “mismatch between input and return,” thereby lowering their experience scores (21, 22). In addition, family members with self-funded medical care may pay more attention to the “practicality” of services due to economic pressure, such as the necessity of examination items and the rationality of drug prices (23). If medical staff do not fully communicate information related to costs, it may exacerbate their distrust of medical behaviors (24). This suggests that medical services need to focus not only on the technical level but also on the transparency of cost communication to alleviate the economic anxiety of self-funded patients.

Based on our findings, clinical nursing and management can be optimized collaboratively from multiple dimensions to improve service quality: aiming at the core issue of the low score in the “medical process” dimension, a “one-stop service center for pediatric infusion” can be established to integrate functional modules such as registration, payment, and appointment for examinations (25, 26); at the same time, information-based means such as intelligent queuing systems and real-time push of examination progress can be used to shorten family members’ perceived waiting time, and an “artificial + intelligent” dual-track service model can be specifically designed for elderly family members to bridge the gap in their technical adaptability (27). For family members with lower educational levels, visual methods such as graphic manuals and video demonstrations can be used to simplify the explanation of medical processes and precautions (28); for family members of first-born and younger children, “one-on-one” care guidance, covering practical content such as comfort skills and key observation points during infusion, should be provided to effectively reduce their anxiety levels (29). In terms of caring for special groups, a “fee consultation window” can be set up for self-funded patients to systematically explain the details of charging items and medical insurance alternative plans; at the same time, volunteers can be assigned to assist elderly family members in completing information-based operations to reduce negative experiences caused by operational obstacles. In addition, a “mother-infant comfort area” can be set up in the infusion room with facilities such as toys and picture books to alleviate the discomfort of younger children (30); medical staff need to strengthen empathic communication with family members, especially taking the initiative to explain the necessity and rationality of medical behaviors to family members of first-born children to enhance their trust in medical services. These measures may systematically improve the medical experience of family members of children in the pediatric infusion room by precisely targeting key influencing factors, thereby providing practical support for building a harmonious doctor-patient relationship.

Our study has several limitations that merit consideration. First, as a single-center investigation, our findings may be influenced by population-specific and regional characteristics, which could limit their generalizability to other healthcare settings or demographics. Second, our regression model explains 56.6% of the variance in the dependent variable, with 43.4% remaining unexplained. While this *R*^2^ value is considered acceptable in social sciences and patient experience research—fields where phenomena are inherently multi-determined—it nonetheless indicates that our model captures only a portion of the complex factors influencing outcomes. The unexplained variance may be attributable to variables not included in our analysis, such as unmeasured individual differences, situational factors, or other contextual influences. Although R-squared can often be increased by adding more predictors, we prioritized theoretical relevance in variable selection and used adjusted R-squared to guard against overfitting. Future research could benefit from incorporating a more comprehensive set of predictors, including patient-reported psychological factors and objective process metrics, and exploring alternative model specifications to further enhance explanatory power. Additionally, our findings should be interpreted in light of the study's sample characteristics and cross-sectional design, which limit the generalizability of results and preclude causal inference.

## Conclusion

In summary, the findings of this study collectively indicate that the medical experience of family members accompanying children in the pediatric infusion room remains suboptimal and warrants targeted improvements to bridge the gap between current service delivery and family expectations. The moderate overall experience score, coupled with the identified influencing factors, underscores the necessity for a proactive approach to enhancing service quality in this high-stakes clinical setting. Notably, the multivariate regression analysis highlights specific subgroups of family members who are more vulnerable to poor medical experiences, including older family members, those with lower educational attainment, caregivers of younger children, families with first-born children, and those relying on self-funded medical insurance. These groups exhibit unique challenges that demand tailored interventions: older family members may face barriers in navigating digital healthcare systems, while individuals with lower educational levels might struggle with understanding complex medical procedures or administrative processes. Caregivers of younger children, particularly infants and toddlers, often experience heightened anxiety due to the child's limited ability to communicate discomfort, compounded by the stress of managing both caregiving responsibilities and healthcare logistics. First-born children's families, typically less experienced in pediatric healthcare interactions, may harbor higher expectations and greater uncertainty, amplifying dissatisfaction when services fall short. Additionally, self-funded families, burdened by direct financial liabilities, are more sensitive to perceived inefficiencies or inadequacies in service, as these directly impact their cost-benefit assessment of care. Thus, healthcare providers and administrators should prioritize these high-risk subgroups when designing quality improvement strategies. By acknowledging their distinct needs and addressing the root causes of their dissatisfaction—such as optimizing service workflows, enhancing communication clarity, and providing targeted support for vulnerable populations—pediatric infusion services may move toward a more patient- and family-centered model, ultimately fostering better experiences, stronger adherence to medical guidance, and more harmonious doctor-patient relationships.

## Data Availability

The original contributions presented in the study are included in the article/Supplementary Material, further inquiries can be directed to the corresponding author.
